# Congenital Melanocytic Nevus Regression Lacking the Halo Phenomenon: The Influence of the Body Site

**DOI:** 10.5826/dpc.1103a28

**Published:** 2021-07-08

**Authors:** Maristela Garcia Bassotto de Andrade, Giuseppe Argenziano, Vincenzo Piccolo, Eugenia Veronica Di Brizzi, Elvira Moscarella

**Affiliations:** 1 Dermoscopy Fellow, University of Campania “L. Vanvitelli”, Naples, Italy; 2 Dermatology Unit, University of Campania “L. Vanvitelli”, Naples, Italy

**Keywords:** Congenital melanocytic nevus, spontaneous regression, children, involution of nevus, pediatric dermatology, dermoscopy

## Introduction

Regression of congenital melanocytic nevus (CMS) has been reported, mainly associated with an immunological mechanism and with the occurring halo phenomenon (a depigmentation zone appearing around the nevus). According to Tokura et.al (as cited in Kageshita, 2003) CD8 T-cell mediated immunity and IgM antibodies may be involved in immunological mechanism of regression [1]. The spontaneous or idiopathic regression of a congenital nevus is uncommon and seems to be most frequent when localized at the level of the palmoplantar region [1,2].

## Case Presentation

**Figure 1:**
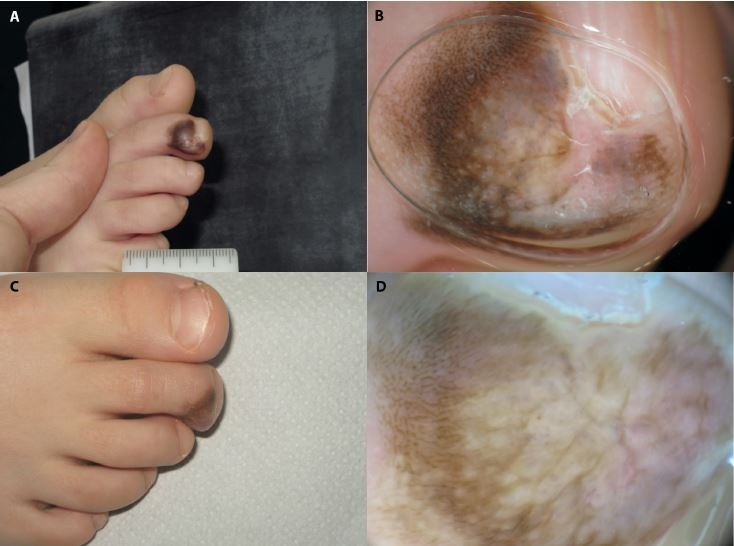
(A) Brownish papule with a heterogeneous color measuring about 1 cm located on the second toe of the right foot. (B) On dermoscopy, the lesion showed a reticular and a parallel furrows pattern at the periphery. In the central-nodular region, multiple grayish dots and globules over a pink-white background were visible. (C) Clinical lightening of the lesion after 3 years. (D) The lesion appeared cleared in color, both peripherally and in the center.

Here we report a case of a 6-year-old male, presenting a lesion on the second finger of the right foot that appeared at birth. Clinically, it presented as a 1cm diameter nodule, characterized by a heterogeneous brownish color, a hypo-pigmented raised area at the center, and well-defined borders (Figure 1A). When analyzed through dermoscopy, the lesion showed a peripheric reticular pattern with parallel furrows pattern visible on 1 side of the lesion. In the central-nodular area, the presence of multiple grayish dots and globules over a pink-white background was detected (Figure 1B).

The lesion was monitored over 3 years. It did not vary in size, but underwent dramatic pigmentation changes (Figure 1C), turning color to light brown, both at the periphery and in the center (Figure 1D).

## Conclusion

CMN may regress in a variety of ways. Ciampo et al. [2] described several distinct mechanisms including trauma, the presence of the depigmentation halo, desmoplasia or even regression without any of the mentioned modalities, which was called idiopathic.

Although idiopathic regression is uncommon, Kageshita (2003) [1], reported on 2 cases of complete CMN spontaneous regression, in the palmar region of 2 Japanese girls.

Despite the absence of a histopathological confirmation, the author concluded that there might be a regression mechanism unrelated to autoimmune issues. According to the statistical analysis carried out by Ciampo et al [2], it was observed that the probability of regression in a congenital nevus located in the palmar and plantar region is 4.95 times greater than in other locations.

Our patient presented a clearing of the lesion color with no evidence of other mechanisms, suggesting a stop in pigment production by the nevus as a regression mechanism, as it occurred in the above-mentioned cases. Moreover, the location of the lesion supports the hypothesis that the palmoplantar location is relevant in the spontaneous regression process. Our report lacks histopathologic examination results, therefore larger case series supported by histopathologic examination are necessary.
